# Pseudogene SNRPFP1 derived long non-coding RNA facilitates hepatocellular carcinoma progress in vitro by sponging tumor-suppressive miR-126-5p

**DOI:** 10.1038/s41598-022-24597-5

**Published:** 2022-12-19

**Authors:** Nan Wang, Simin Guo, Fengjie Hao, Yifan Zhang, Yongjun Chen, Xiaochun Fei, Junqing Wang

**Affiliations:** 1 Department of General Surgery, Ruijin Hospital, Shanghai Jiao Tong University School of Medicine, 197, Rui Jin Er Road, Shanghai, 200025 People’s Republic of China; 2 Department of Infectious Disease, Ruijin Hospital, Shanghai Jiao Tong University School of Medicine, 197, Rui Jin Er Road, Shanghai, 200025 People’s Republic of China; 3 Department of Pathology, Ruijin Hospital, Shanghai Jiao Tong University School of Medicine, 197, Rui Jin Er Road, Shanghai, 200025 People’s Republic of China

**Keywords:** Cancer, Biomarkers

## Abstract

Pseudogene-derived transcripts, especially those barely transcribed in normal tissues, have been regarded as a kind of non-coding RNAs, and present potential functions in tumorigenicity and tumor development in human beings. However, their exact effects on hepatocellular carcinoma (HCC) remain largely unknown. On basis of our previous research and the constructed online database for the non-coding RNAs related to HCC, a series of pseudogene transcripts have been discovered, and SNRPFP1, the homologous pseudogene of SNRPF, was found to produce an anomalously high expression long non-coding RNA in HCC. In this study, we validated the expression of the SNRPFP1 transcript in both HCC tissues and cell lines. The adverse correlation between SNRPFP1 expression and patients’ outcomes was observed. And depletion of SNRPF1 in HCC cells significantly suppressed cell proliferation and apoptosis resistance. Meanwhile, the motility of HCC cells was potently impaired. Interestingly, miR-126-5p, one of the tumor-suppressive genes commonly decreased in HCC, was found negatively expressed and correlated with SNRPF1, and a specific region of SNRPF1 transcript is directly binding to miR-126-5p in a molecular sponge way. The rescue experiment by knock-out miR-126-5p significantly reversed the cell growth suppression and a higher ratio of cell apoptosis induced by SNRPF1 depletion. Lastly, we concluded that SNRPF1 is a pseudogene active in HCC, and its abnormally over-expressed transcript is a strong promoter of HCC cell progress in vitro by sponging miR-126-5p. We believe that the findings in this study provide new strategies for HCC prevention and therapeutic treatment.

## Introduction

Hepatocellular carcinoma (HCC) ranks third in the world of tumor-related mortality, which gives out great challenges to figuring out better prognoses and outcomes for HCC patients^1,2^. In recent years, despite the impressive progress in HCC treatment strategies, like targeted therapy and tumor immunotherapy, the overall survival of HCC patients is not satisfactory^3^. It is important to discover innovative and practical therapeutic targets for efficient methods of preventing and treating HCC.

To date, a large number of transcripts in different RNA species generated from the human genome have been discovered, and the main part of the total transcripts are sequences that lack the capacity for protein coding. Generally, two major classes of the non-coding RNAs (ncRNAs) were set up according to the size of the transcripts, the small non-coding RNAs that are composed of less than 200 nucleotides such as the acknowledged microRNAs (miRNAs), and the long non-coding RNAs (lncRNAs), composed of over 200 nucleotides^4,5^.

Accumulating evidence has revealed that lncRNAs are the majority of ncRNAs, and are widely expressed in human beings, playing key roles in gene regulation. The functions of lncRNAs are complex depending on the specific localization of different lncRNAs intracellular and their particular modes of interactions with DNA, RNA, and proteins^6^. In brief, even the exact mechanisms of the functional lncRNAs are still debated and controversial, the effects of lncRNAs involve a variety of regulation events including gene transcription, mRNA translation, protein modification, and the formation of RNA–protein or protein–protein complexes^7^.

Pseudogenes are special DNA sequences generated from their parental protein-coding genes via acquired mutation and duplication^8,9^. Pseudogenes are commonly profiled with the characteristic of lacking promoter and premature stop codon, or frameshift mutation^10^. Based on this, transcription is limited in most of the pseudogenes. However, recent research illustrated that pseudogenes could be transcribed pathologically and participate in specific functions, like gene expression modulation through the miRNA decoy effect, or exert either tumor-suppressive or oncogenic functions^11,12^. And notably, transcripts of these pseudogenes are commonly non-coding RNA sequences with over 200 nucleotides in length, which belong to the category of lncRNAs. Thus, even though the knowledge of the pseudogene effects on human malignancies is in limitation and confusing, we can still make sense of their important roles through the lncRNA-related functions, for example, the competitive endogenous RNA (ceRNA) effect or sponge-like effect.

Theoretically, miRNAs bind to the gene transcripts containing the miRNA-response elements (MREs), which are specific sequences on the transcripts and are recognized and interact with miRNAs. CeRNAs could bind to corresponding miRNAs through the MREs with high affinity and sequentially abrogate the interaction and degeneration effects of miRNAs on the downstream targeting mRNAs. No doubt, a series of pseudogene transcripts potentially carry the MREs, and some of them had been discovered to exert the ceRNA effect in human cancers^13^. For instance, PTENP1, which was the first classic pseudogene, suppressively impacts the progress of tumors, such as gastric cancer and clear cell renal carcinoma, by protecting its parental gene PTEN through a ceRNA effect binding with miR-21 or miR-19^14,15^. On the contrary, in a similar ceRNA way, pseudogene BRAFP1 transcript exerts tumor promoter by sponging particular miRNAs to preserve the expression of its parental gene BRAF and sequentially leads to the activity of the downstream oncogenic MAPK pathway in lymphoma^16^. Consistent with these reports, our previous research has demonstrated an efficient ceRNA effect induced by AKR1B10P1, a pseudogene of AKR1B10 barely transcribed in the normal liver, on tumor-suppressive miR-138 and sequentially promotes HCC tumor growth^17^. All these findings above provide us with new possibilities for breakthroughs in exploring the complex mechanism of HCC progress.

In our recent study, we have successfully constructed and reported an online database with the analysis tools, named the Liver Cancer lncRNA Explore (LCLE) online tool (https://datasciences123.shinyapps.io/LCLE/), on basis of the random walk-based multi-graphic (RWMG) model algorithm developed by our team. And it is used for comprehensive and precise analysis and screening of HCC-related lncRNAs and the relative networks and pathways, including the pseudogene transcripts^18^. According to these preliminary works, we discovered that Small Nuclear Ribonucleoprotein Polypeptide F Pseudogene 1 (SNRPF pseudogene 1, SNRPFP1) is anomalously activated in transcription. And by detecting the expression profile of SNRPFP1 along with its parental gene SNRPF, we found an adverse correlation between SNRPFP1 and patients’ outcomes and were commonly inclined to dismal clinicopathological features in the cases collected from our medical center. Depletion of SNRPFP1 transcript in vitro significantly impaired the HCC cells’ abilities on cell proliferation, apoptosis resistance, and cell motility. Meanwhile, we noticed that miR-126-5p was significantly decreased in HCC cells and negatively correlated with SNRPFP1 transcript expression. Furtherly, we validated the ceRNA effect of SNRPFP1 transcript on miR-126-5p. And by modulating miR-126-5p expression for the rescue experiment, we verified that pseudogene SNRPFP1 is dysregulated in HCC and transcriptional activated, through which it facilitates HCC progress in *vitro* by directly sponging miR-126-5p. Thus, we prompt that these findings point out hopeful targets for HCC control in an AS-related way.

## Materials and methods

### Cell culture

Three typical HCC cell lines (Huh7, HepG2, and Hep3B) were recruited in this study, and the normal human hepatic cell LO2 was used as a control (Shanghai Institutes for Biological Sciences, Chinese Academy of Science, Shanghai, China)). All the cells were cultured by RPMI 1640, supplemented with 10% heat-inactivated fetal bovine serum (FBS), incubated at 37 °C environment temperature, with 100 ug/ml streptomycin, and 100U/ml Penicillin in a humidified cell, with an atmosphere of 5% CO_2_. Specifically, for the transfected cells, a medium mixed with G418 (Santa Cruz Biotechnology, Inc; 400 μg/ml) was used for selection.

### Preparation of the pathological specimens

Eighty-seven paired specimens including the tumor tissue and the adjacent non-cancerous liver tissues were collected from the patients diagnosed and conducted radical resection with no preoperative treatment, in 2016 –2019, at the Department of General Surgery, Ruijin Hospital, Shanghai Jiao Tong University School of Medicine. The corresponding clinicopathologic parameters of the patients were obtained including gender, age, tumor size, number of lesions, grades et.al. Informed consent was obtained, and the study was approved by the Ethics Committee of Ruijin Hospital, Shanghai Jiao Tong University School of Medicine. All methods were performed following the relevant guidelines and regulations.

### Datasets preparation

The liver cancer datasets were collected from the Cancer Genome Altas database (TCGA: https://tcga-data.nci.nih.gov/tcga/) containing gene profiles of 369 HCC tumor samples and 50 paired adjacent non-cancerous samples.

The gene expression data for 110 normal liver samples from the GTEx and TCGA along with clinical information for 369 liver tumors and 50 normal samples from the UCSC Xena database (https://xenabrowser.net/datapages/) were intensively explored by using the RWMG model algorithm we reported, and the related and comprehensive information of the involved genes was described in the Liver Cancer lncRNA Explore (LCLE) online tool (https://datasciences123.shinyapps.io/LCLE/), constructed by the collaborators of our team. Simultaneously, the starBase datasets (https://starbase.sysu.edu.cn/) and the dreamBase (https://rna.sysu.edu.cn/dreamBase/) datasets were used for providing Supplementary Information on the expression and relationship of the candidate genes in this study.

### RT-qPCR assay and immunohistochemistry assay

RNA isolation, from either tissues or cells, is conducted according to the instruction of the TRIzol reagent (Invitrogen, USA). The first-strand cDNA was synthesized via High-Capacity cDNA Reverse Transcription Kit (ABI, USA). All the primers were synthesized (Jike Biotech Company, Shanghai, China) (Supplementary Table 1). Real-time quantitative polymerase chain reaction (RT-qPCR) was operated following the TaqMan Gene Expression Assays protocol (ABI, USA). The relative quantification of RNA in cell lines was normalized using GAPDH by the 2^*−*Δ*CT*^ method. The expression level of miR-126-5p was measured according to the Taqman^®^ MicroRNA Assays protocol (Applied Biosystems) and normalized by using U6 small nuclear RNA (RNU6B; Applied Biosystems) through the 2^−ΔCT^ method. The relative expression ratio of miR-126-5p in each paired tumor to non-cancerous tissue was calculated by the 2^−ΔΔCT^ method. When the relative expression ratio of miR-126-5p in the tumor was < 1.0, it was defined as a down-regulation; and > 1.0 was defined as an up-regulation. The PCR program was set as follows: 95 °C for 10 min, followed by 35 cycles of 95 °C for 15 s, 60 °C for the 30 s, and 72 °C for 45 s.

Antibodies against SNRPF were prepared (Abcam, USA). The Western blot analysis and immunohistochemistry assay The protein expression levels detected by IHC were assigned to two experienced pathologists who independently follow our previously described methods^19^, for blind examination and were separated into two groups by staining intensity grade: no to low staining (0 ~ 1 +) and moderate to high staining (2 +  ~ 3 +).

### Plasmid construction and transfection

The lentiviral vectors pLKO.1 (Addgene, Cambridge, USA) containing shRNA was transfected into cultured HepG2 and Hep3B cells at the exponential phase (JIKE Biochemistry, Shanghai, China) for suppressing SNRPFP1 transcript expression. The control ones were set up. The transfected cells were selected by using a medium mixed with G418 (Santa Cruz Biotechnology, Inc; 400 μg/ml). The mimic was used to transfect HCC cells for ectopically introducing miR-126-5p (HepG2/miR-126-5p; Hep3B/miR-126-5p), and the negative controls (HepG2/NigmiR; Hep3B/NigmiR) were set. And the rescue experiment by knock-out miR-126-5p was set up by using the siRNA method using the protocol and siRNA vector tools designed by Jike Biotech Company, (Shanghai, China), and the validation was implemented by the RT-qPCR assay.

### Cell proliferation assay and cell cycle analysis

The treated HCC cells (1 × 10^6^) were cultured in 96-well microtiter plates triplicated and incubated at an atmosphere of 5% CO_2_ and 37 °C for 5 days. Microplate computer software (Bio-Rad Laboratories, Inc., Hercules, CA, USA) was applied for measuring the OD following the Cell Counting Kit-8 (CCK-8) assay kit protocol (Dojindo, Tokyo, Japan). Then, we plotted the cell proliferation curves. Meanwhile, the cells were treated with ethanol fixation, followed by RNase A treatment and propidium iodide staining. Flow cytometry detection was carried out using FACSCalibur (Becton–Dickinson, Franklin Lakes, NJ, USA) for quantifying cell populations at the G0/G1, S, and G2/M phases, and ModFit software e (Becton–Dickinson) was used. The debris and fixation artifacts of the cells were excluded.

### Cell apoptosis analysis

Cell apoptosis rate was calculated by using PE-Annexin V Apoptosis Detection Kit I (BD Pharmingen, USA) following the instructions. Transfected cells were resuspended in the concentration of 1×10^6^ cells/ml by the 1×Binding Buffer. 5 μl of FITC and 5 μl of PI were added into 100 μl of the cell suspension, followed by a 15 min incubation in darkness, added with 400μl×Binding Buffer. The apoptosis rate was calculated through flow cytometry (Becton Dickinson, USA). Both Annexin V-FITC-positive and PI-negative cells were considered apoptosis cells.

### Cell motility investigation

The cell invasion and migration capacity were analyzed by using the QCMTM 24-Well Colorimetric Cell Migration or Invasion Assay Kit (Millipore, USA). 3×10^4^ stable transfected HCC cells in 300 ml serum-free medium were added to the upper chamber, and 10% FBS-containing medium was used as a chemoattractant in the lower chamber. ECMatrixTM was precoated to the upper chamber for invasion assay, and cells on the bottom of the membrane were stained and checked in 24 h for migration or in 48 h for invasion.

### Dual-luciferase reporter assay

BY using the online tools of the dreamBase (http://rna.sysu.edu.cn/dreamBase/), miR-126-5p was respectively predicted directly binding to the transcript of SNRPFP1. The potential specifical binding sequence was intercepted for the 202 bp sections along with the corresponding mutative ones for further detection by the dual-luciferase reporter assay. (Supplementary Table 2). And the sequence was cloned into the pMIR-Report luciferase vector, which contains firefly luciferase, and the pRL-TK vector luciferase was set as control (Promega, Madison, WI, USA). These two sets of vectors were co-transfected into both HepG2 and Hep3B cells transfected with miR-126-5p mimics or the control ones. The luciferase activity was measured via the Dual-Glo Luciferase assay system (Promega) 48 h after the transfection.

### Statistical analysis

As a statement, we declare that all methods were performed following the relevant guidelines and regulations. The statistical analysis was carried out by using SPSS 20.0. *P*-values were calculated using an unpaired Student’s *t* test and Fisher’s exact test. Differences were considered statistically significant at *P*-values < 0.05.

### Ethics approval and consent to participate

Informed consent was obtained and the study was approved by the Ethics Committee of Ruijin Hospital, Shanghai Jiaotong University School of Medicine, following the Declaration of Helsinki.

## Results

### Pseudogene SNRPFP1 activated to highly transcribe in HCC cell lines and tissues

**Figure 1:**
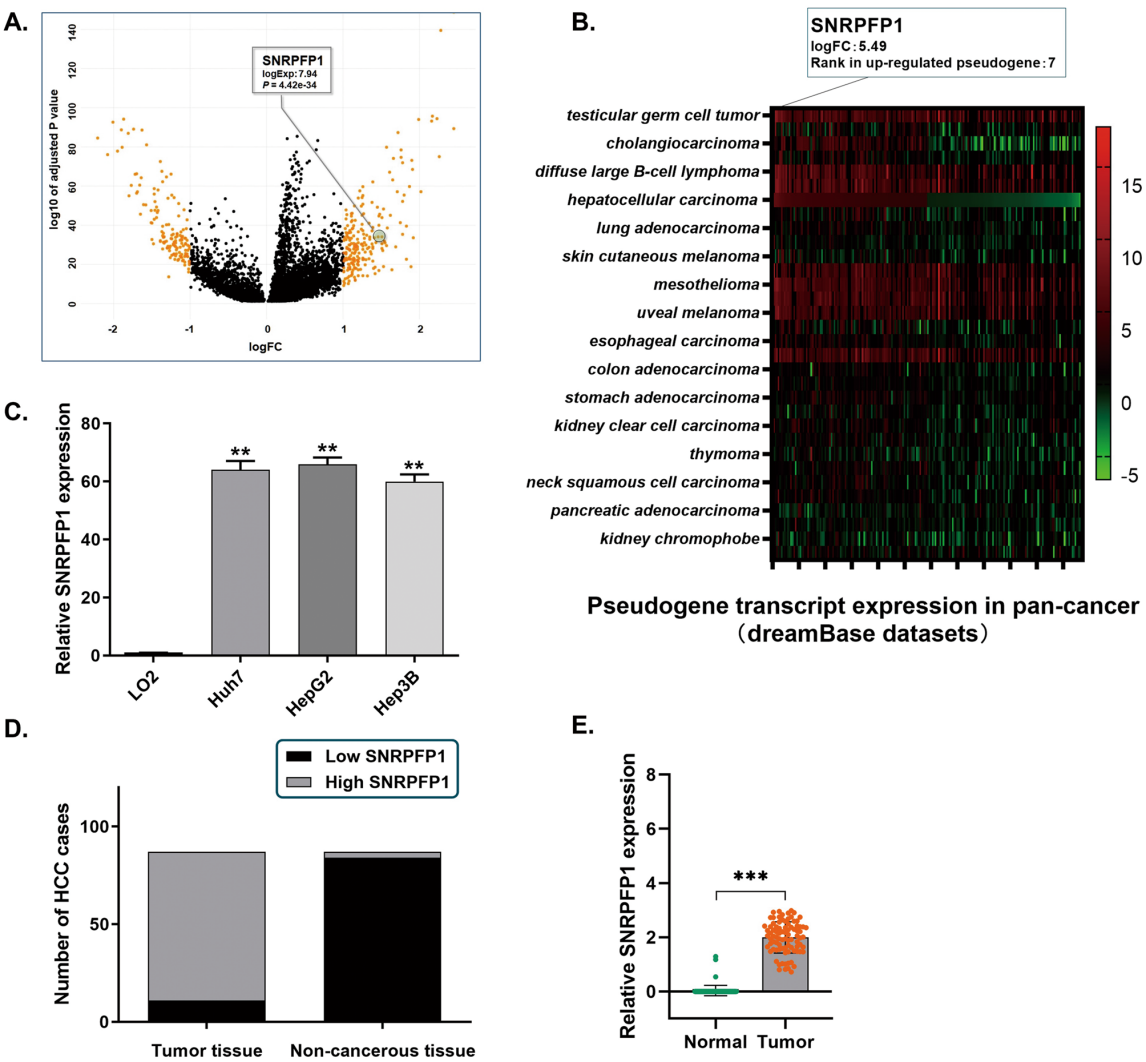
Pseudogene SNRPFP1 activated to highly transcribe in HCC cell lines and tissues. (**A**) Analysis of the data from the LCLE. Pseudogene SNRPFP1is transcriptionally activated and presents a high expression in HCC. (**B**) Analysis of the dreamBase datasets, The heatmap indicated the expression profile of the transcript of SNRPFP1 in pan-cancer. (**C**) RT-qPCR assay was carried out to demonstrate the SNRPFP1 transcript level in HCC cell lines, compared with LO2 cells. SNRPFP1 transcript level in LO2 cells was extremely low and indicated that the SNRPFP1 gene was barely transcribed. On the contrary, a significantly high expression of SNRPFP1 transcript in three HCC cell lines was observed, which indicated that the SNRPFP1 gene was remarkably transcribed in HCC cells (**P < 0.01). (**D**) Statistic of the number of cases concerning the expression of SNRPFP1 transcript in HCC specimens. SNRPFP1 transcript is detectable and highly expressed in most of the tumor tissues (76/87), and was not detected in most of the adjacent non-cancerous tissues (84/87) (*P* < 0.01). (**E**) RT-qPCR assay was conducted in the 87 real patients’ specimens. The SNRPFP1 transcript was significantly highly expressed in tumor tissues, and only very few detectable SNRPFP1 transcript was found in the non-cancerous tissues (**P < 0.01).

By exploring and analyzing the data from the LCLE and the dreamBase datasets, we obtained the expression profile of the transcript of SNRPFP1 in either pan-cancer or HCC (Fig. 1A,B). As observed, SNRPFP1 presents merely no expression in normal organs or tissues, including the liver. However, in multiple malignancies, SNRPFP1 shows an elevation in transcription. And for HCC, a remarkable ascent of SNRPFP1 transcript has been demonstrated.

On basis of the results of the databases, the expression of SNRPFP1 transcript in three HCC cell lines (Huh7, HepG2, and Hep3B) was detected by RT-qPCR assay. The level of SNRPFP1 transcript in three HCC cell lines is significantly higher than in the control LO2 cells (Fig. 1C). And accordingly, in real patients’ specimens, a high expression profile of SNRPFP1 transcript was verified in HCC tumor samples, compared with the adjacent non-cancerous liver tissues. As shown in Fig. 1D–E, the SNRPFP1 transcript is detectable in all of the tumor specimens, and 87.35% (76/87) HCC specimens presented obvious SNRPFP1 transcript expression, and only 12.64% (11/87) tumor tissues presented a relatively lower level but detectable SNRPFP1 transcript. In contrast, 3.45% (3/87) of the adjacent non-cancerous liver tissues presented detectable low expression of SNRPFP1 transcript, and the others were reported with no detectable SNRPFP1 expression. Interestingly, we also detected the expression of the parental gene SNRPFP1 by RT-qPCR assay and the IHC assay. And as expected, 82.76% (72/87) of tumor specimens presented highly SNRPF mRNA expression along with a strong positive expression through the IHC assay. On the contrary, only 13.79% (12/87) of the adjacent liver specimens showed relatively higher SNRPF expression (Supplementary Fig. 1). Thus, we suppose that SNRPFP1 transcription is activated in HCC and co-expressed with its parental gene.

### SNRPFP1 transcript is correlated with the HCC patients’ clinicopathologic features

The correlation between SNRPFP1 transcript in HCC tissues and the clinicopathologic features of the 87 HCC cases was analyzed statistically. As Table 1 show, that there is no significant correlation between SNRPFP1 and the patient’s age, gender, and virus control status. Whereas, the transcriptional activation of SNRPFP1 in tumor tissues shows an adverse relationship with the tumor size (*P* < 0.05), serum Alpha-fetoprotein (AFP) quantity (*P* < 0.05), frequency of advanced TNM stages (*P* < 0.05), tumor microsatellite formation (*P* < 0.05), invasion of venous (*P* < 0.05), and liver cirrhosis stages (*P* < 0.05). These findings above suggest that SNRPFP1 transcriptional events may play important role in HCC. Similar results were obtained between the parental gene SNRPFP1 and the clinicopathologic features (Supplementary Table 3).

**Table 1:** Correlation between SNRPFP1 transcript and clinicopathological features in 87 HCC specimens.

Clinicopathological parameters	SNRPFP1 transcript level		P*
	Low (n = 11)	High(n = 76)	
**Age (years)**			
≤ 50	7	39	0.529
> 50	4	37	
**Gender**			
Male	8	38	0.205
Female	3	38	
**Diameter (cm)**			
≤ 5	9	34	0.026
> 5	2	42	
**TNM stage**			
I –II	8	24	0.016
III – IV	3	52	
**Tumor encapsulation**			
Absent	7	29	0.188
Present	4	47	
**Tumor microsatellite formation**			
Absent	8	29	0.048
Present	3	47	
**Venous invasion**			
No	6	26	0.203
Yes	5	50	
**HBsAg**			
Negative	2	9	0.625
Positive	9	67	
**AFP(ng/ml)**			
≤ 400	6	14	0.016
> 400	5	62	
**Cirrhosis**			
Absent	4	6	0.020
Present	7	70	

SNRPFP1 transcript level associated with clinicopathologic features in 87 HCC patients, including age, gender, tumor size, tumor stage (AJCC), tumor encapsulation, tumor microsatellite formation, vein invasion, HBsAg status, AFP level, and liver cirrhosis. Statistically, significance was assessed by Fisher’s exact test.

### Depletion of SNRPFP1 represses cell proliferation in HCC cells and arrests cell cycles

**Figure 2:**
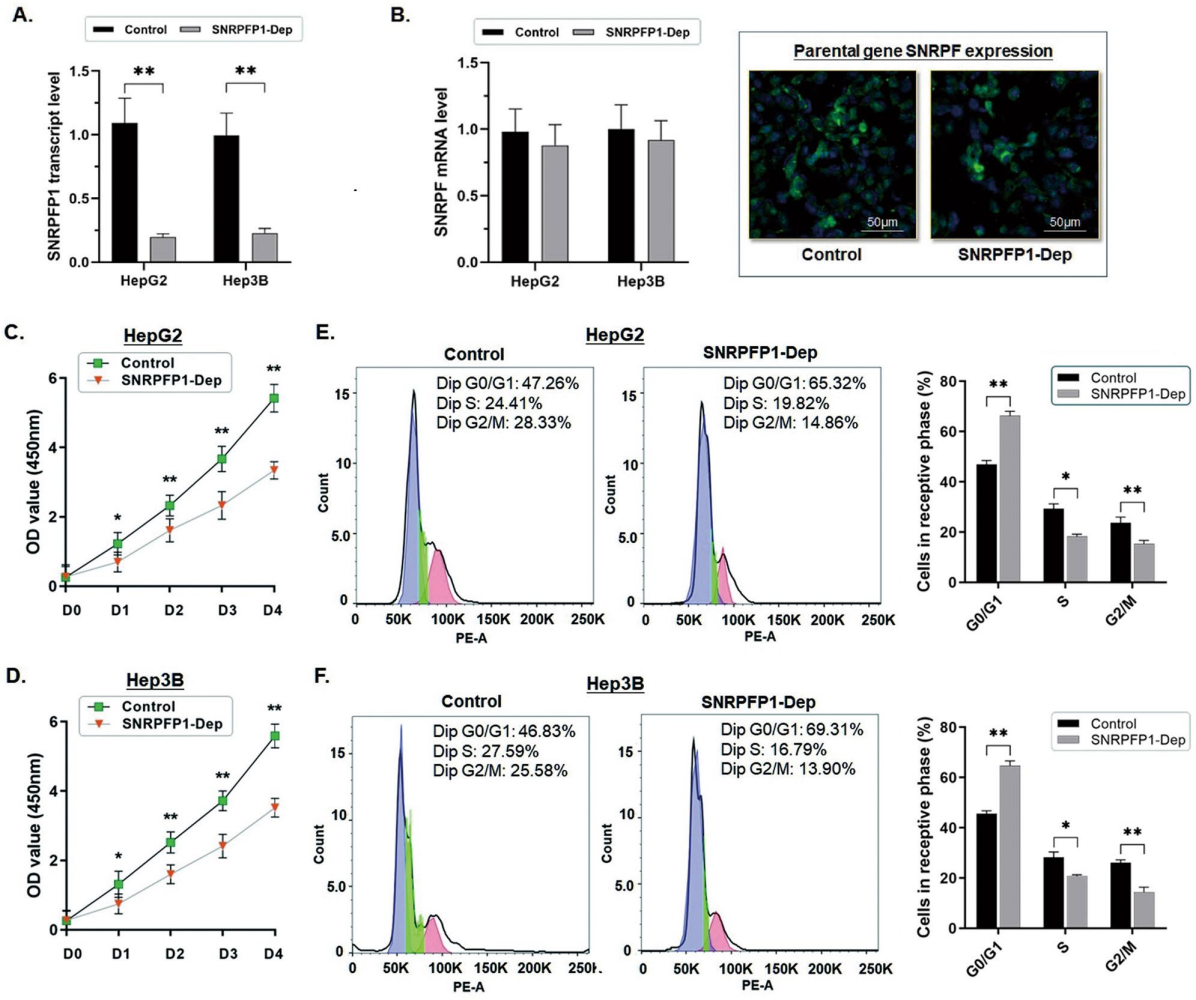
Depletion of SNRPFP1 represses cell proliferation in HCC cells and arrests cell cycles. (**A**) RT-qPCR assay was carried out to determine the effect of expression depletion on SNRPFP1 transcript in HCC cells. Depletion of SNRPFP1 transcript was conducted in HepG2 and Hep3B cells by using shRNA transfection. The RT-qPCR assay demonstrated the significant abrogation of SNRPFP1 transcript in both HepG2 and Hep3B cells (**P < 0.01). (**B**) The RT-qPCR assay and immunofluorescence were conducted to determine the change in the expression of the parental gene SNRPF when SNRPFP1 was depleted in HepG2 and Hep3B cells. No significant change in SNFPF expression was induced when the SNRPFP1 transcript was depleted. And the green fluorescent density indicating SNRPF expression in HCC cells made no significant change in comparison with the control. (**C,D**) The Flow cytometry detection of the cell cycle demonstrated that when SNRPFP1 transcript was abrogated in either HepG2 or Hep3B cells, the ability of cell proliferation was significantly impaired (*P < 0.05; **P < 0.01). (**E,F**) Flow cytometry was used for analyzing the cell cycle. The representative histograms demonstrate the cell cycle status in HepG2 and Hep3B cells. The cell cycle of both HepG2 and Hep3B cells was significantly arrested in G0/G1 phase by depleting the SNRPFP1 transcript. The results are means of three independent experiments ± SD. (*P < 0.05, **P < 0.01).

HepG2 and Hep3B cells were transfected with shRNA against SNRPFP1 transcript, and the RT-qPCR assay was carried out to validate the effect on SNRPFP1 depletion, and the expression of the parental gene was not affected (Fig. 2A,B). The cell proliferation was significantly suppressed in both HepG2 and Hep3B cells when SNRPFP1 transcription was abrogated (Fig. 2C,D). (*P < 0.05; **P < 0.01).

The flow cytometric analysis demonstrated that the cell cycle of the HCC cells was significantly arrested at the G0/G1 phase along with SNRPFP1 depletion (Fig. 2E,F). The percentage of the HepG2 and Hep3B cells in the G0/G1 phase was increased respectively from 45.61 to 64.78% (*P* < 0.01) and from 46.97 to 66.37% (*P* < 0.01). Meanwhile, the obvious percentage decrease was observed in the S phase (HepG2: from 28.28 to 20.79%, *P* < 0.01; Hep3B: from 29.31 to 18.43%, *P* < 0.05) and the G2/M phase (HepG2: from 26.11 to 14.43%, *P* < 0.01; Hep3B: from 23.72 to 15.20%, *P* < 0.01).

### Depletion of SNRPFP1 impairs HCC cell apoptosis resistance and cell mobility

**Figure 3:**
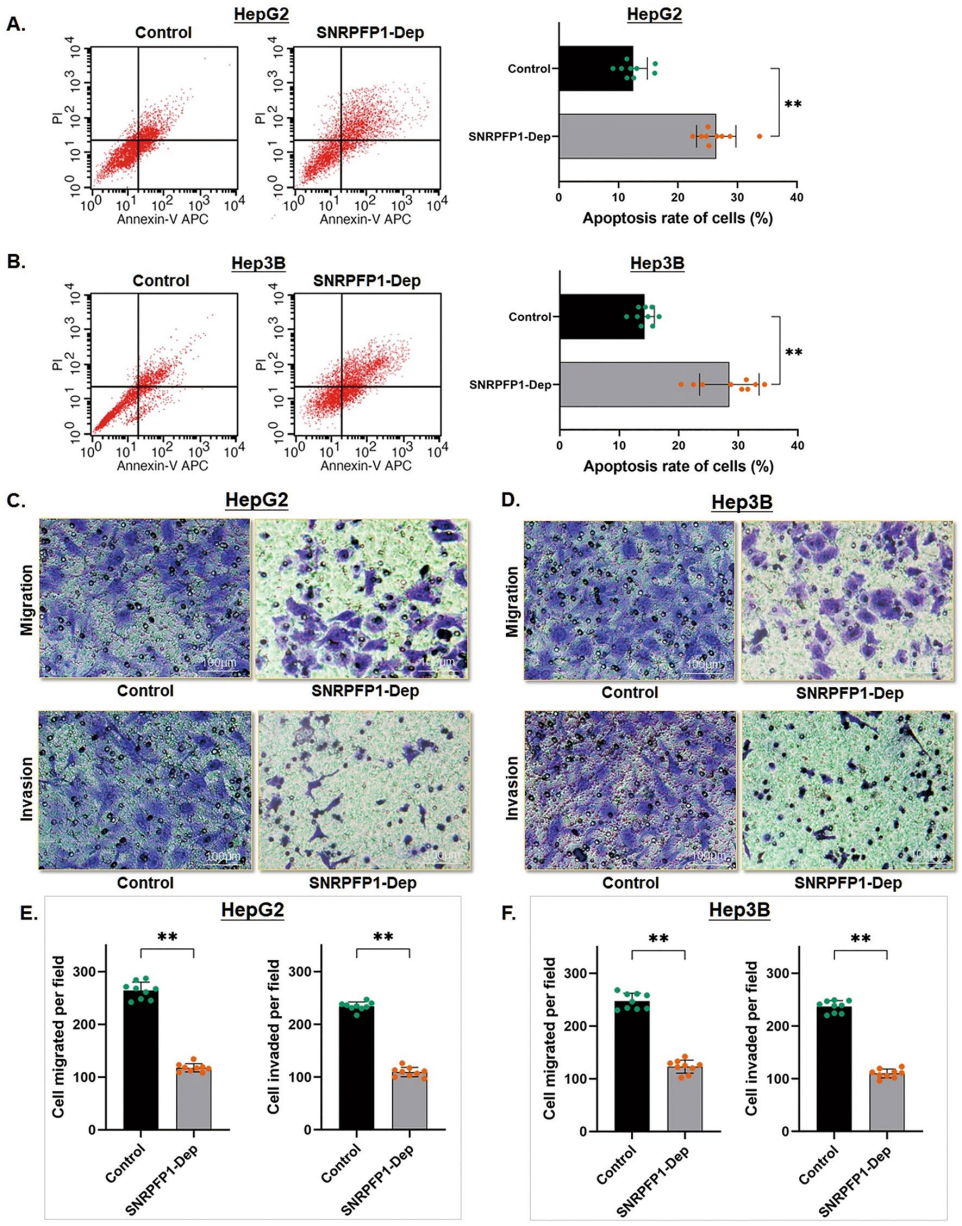
Depletion of SNRPFP1 impairs HCC cell apoptosis resistance and cell mobility. (**A,B**) Cell apoptosis was detected by flow cytometry. The representative histograms demonstrate the apoptosis rate in HepG2 and Hep3B cells. The counting number of apoptotic cells was significantly increased. The results are means of three independent experiments ± SD. (**P < 0.01). **(C,D**) Cell mobility of HepG2 and Hep3B cells was detected through the Transwell assay. As shown the representative histograms of the migration and invasion assay in these two HCC cells respectively. (**E,F**) SNRPFP1 depletion induced a significantly decreased number of cells migrated into the low chamber in both of the two HCC cells (**P < 0.01). Similarly, SNRPFP1 depletion induced a significantly decreased number invaded into the low chamber through the ECMatrixTM pre-coated in both of the two HCC cells. The results are means of three independent experiments ± SD. (**P < 0.01).

The flow cytometric analysis was used for calculating the cell apoptosis rate. Both the cell apoptosis rates in HepG2 and Hep3B cells were significantly increased (HepG2: from 12.49 to 26.43%, *P* < 0.01; Hep3B: from 14.25 to 28.49%, *P* < 0.01) sequentially followed SNRPFP1 depletion, which suggests an enhancement of cell apoptosis resistance induced by SNRPFP1 transcript in HCC cells (Fig. 3A,B).

Along with these changes, the transwell assay also gave out a significant down-regulation of cell mobility in both cell migration and invasion, by depleting SNRPFP1. As shown by the migration assay, the number of HCC cells migrated into the low chamber decreased remarkably after SNRPFP1 depletion (Control/SNRPFP1-Dep: in HepG2 cells as 263 ± 16/117± 9 cells per field, *P* < 0.01; in Hep3B cells as 246± 15/122 ±12 cells per field, *P* < 0.01). As the same, the invasion assay demonstrated that the invaded number of the HCC cells in the low chamber was decreased significantly by depleting SNRPFP1 (Control/SNRPFP1-Dep: in HepG2 cells as 234 ±11/109 ± 11 cells per field, P < 0.01; in Hep3B cells as 236 ± 11/111 ± 9 cells per field, P < 0.01). (Fig. 3C–F). The findings above strongly suggest that the SNRPFP1 transcript could remarkably affect HCC cell mobility and facilitates tumor invasiveness and metastasis.

### SNRPFP1 transcript negatively regulates miR-126-5p in HCC cells through its ceRNA effect

**Figure 4:**
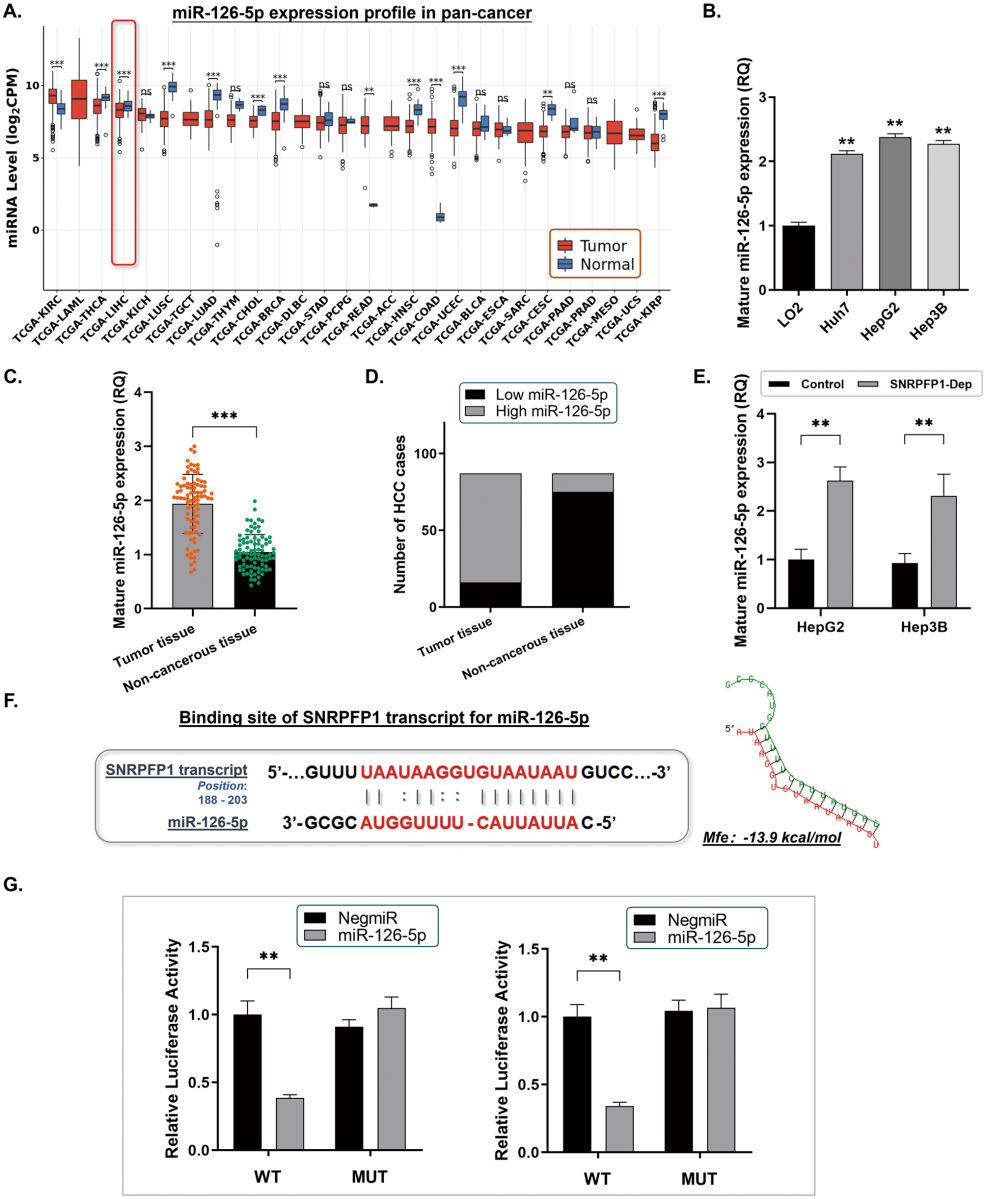
SNRPFP1 transcript negatively regulates miR-126-5p in HCC cells through its ceRNA effect. (**A**) The comprehensive expression profile of miR-126-5p in pan-cancers (**P < 0.001). (**B**) The RT-qPCR assay was carried out to detect the level of miR-126-5p expression. In comparison with the control LO2 cells, all three HCC cell lines presented significantly highly expressed miR-126-5p (**P < 0.01). (**C**) RT-qPCR assay was conducted in the 87 real patients’ specimens. The expression of miR-126-5p was significantly lower in tumor tissues, compared with the non-cancerous tissues (**P < 0.01). (**D**) Statistic of the number of cases concerning the expression of miR-126-5p in HCC specimens. The expression of miR-126-5p is significantly decreased in most of the tumor tissues (7187), and only a small portion of the adjacent non-cancerous tissues was detected with relatively higher miR-126-5p expression (16/87) (*P* < 0.01). (**E**) By using the RT-qPCR assay to detect the HepG2 and Hep3B cells with SNRPFP1 depletion, a significant increase of miR-126-5p expression was observed in both of the two HCC cell lines (**P < 0.01). (**F**) Predicted binding sequence of SNRPFP1 transcript with the seed sequence of miR-126-5p. The minimum free energy (Mfe) hybridization is calculated as: − 13.9 kcal/mol. (**G**) The direct interaction between SNRPFP1 transcript and miR-126-5p was checked by the dual-luciferase reporter assay. Both the HepG2 and Hep3B cells were transfected with the predicted binding site either wildtype (WT-binding site) or mutated (MUT-binding site). MiR-126-5p was up-regulated in these cells through mimics. Up-regulation of miR-126-5p significantly reduced the luciferase signal of these two HCC cells of the WT-binding site (HepG2 or Hep3B/miR-126-5p), compared with the negative control (NigmiR); And, the binding site mutated HepG2 and Hep3B cells suppressed this signal reduction effect (**P < 0.01).

By analyzing the liver cancer datasets of TCGA and starBase, we noticed that miR-126-5p was remarkably decreased in HCC tumor tissues (Fig. 4A). Considering that miR-126-5p plays tumor-suppressive roles in multiple cancers, we also detected miR-126-5p levels in the three HCC cell lines and the 87 HCC samples. As we observed, miR-126-5p was significantly remaining at a low level in comparison with the control LO2 cells (Fig. 4B). Similarly, miR-126-5p was found in most of the HCC tumor tissues (71/87 in tumor vs 16/87 in adjacent non-cancerous tissues) (Fig. 4C,D). And interestingly, when SNRPFP1 was depleted, the miR-126-5p level in the HCC cells was significantly up-regulated (Fig. 4E). Thus, we wondered if there exists a direct regulation between SNRPFP1 transcript and miR-126-5p.

By exploring the transcript of SNRPFP1, we found that a sequence from 188 to 203 bp to the 3’ end of SNRPFP1 transcript is a mighty binding site specifical for miR-126-5p, which prompts a potential ceRNA effect of SNRPFP1 transcript by sponging miR-126-5p (The minimum free energy, Mfe: -13.9 kcal/mol) (Fig. 4F). Based on this, the mutated putative binding site on the SNRPFP1 transcript was constructed for the dual-luciferase reporter assay to further verify the direct interaction between the SNRPFP1 transcript and miR-126-5p. The results illustrated that the luciferase signal in either HepG2 or Hep3B cells transfected with miR-126-5p mimics was significantly decreased, when the cells were transfected with the SNRPFP1/pMIR/WT vector, in comparison with the control cells. On the contrary, the transfection of the SNRPFP1/pMIR/MUT vector induced no significant signal changes (Fig. 4G). Thus, the findings here are convincible to present a sponge-like effect of SNRPFP1 transcript on miR-126-5p.

### Modulating miR-126-5p reverses the phenotypes of HCC cell growth induced by SNRPFP1

**Figure 5:**
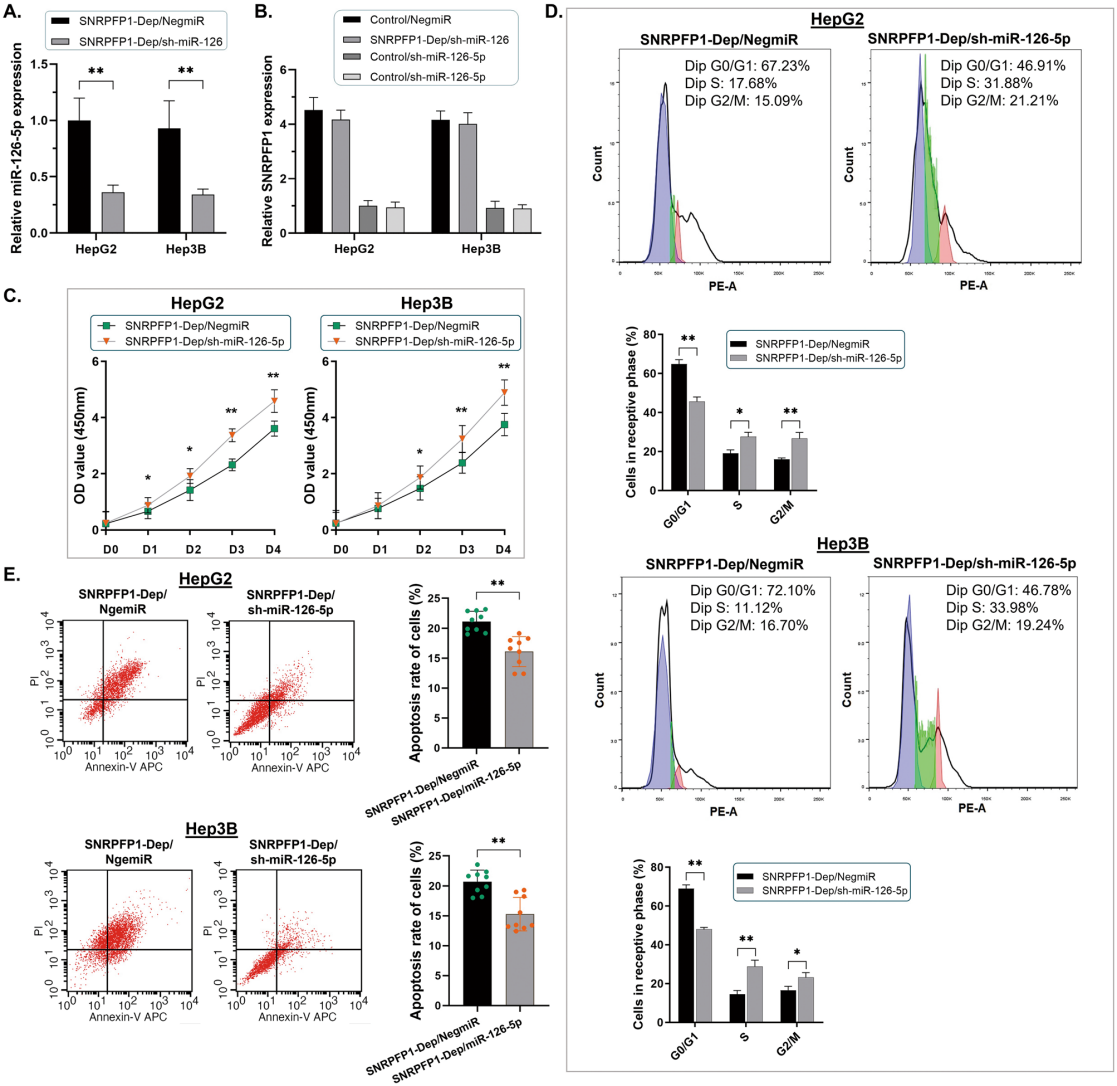
Modulating miR-126-5p reverses the phenotypes of HCC cell growth induced by SNRPFP1. (**A**) The RT-qPCR assay was conducted. The expression of miR-126-5p was knocked out in the SNRPFP1-depleted cell lines of both HepG2 and Hep3B cells (**P < 0.01). (**B**) The RT-qPCR assay indicated no significant change in the expression of the SNRPF gene when miR-126-5p was depleted in the HepG2 and Hep3B cells. (**C**) The suppression of cell proliferation in HCC cell lines treated with SNRPFP1 depletion was significantly rescued when miR-125-5p was knocked out (*P < 0.05; **P < 0.01). (**D**) The Flow cytometry detection of the cell cycle demonstrated that when miR-126-5p was knocked out, the arrested cell cycle induced by SNRPFP1 depletion in either HepG2 or Hep3B cells was reversed (*P < 0.05; **P < 0.01). (**E**) The Flow cytometry detection for counting the cell apoptosis demonstrate a significant decrease in apoptotic cells when miR-126-5p was declined in the HepG2 and Hep3B cells treated with SNRPFP1 depletion (**P < 0.01).

According to the findings that the phenotypes of HCC cell growth were suppressed via depleting SNRPFP1, we conducted the rescue experiment by knock-out miR-126-5p to make sure that the miR-126-5p is the downstream effector of SNRPFP1 in HCC progress (Fig. 5A). As Fig. 5B shows, when the miR-126-5p expression declined in either HepG2 or Hep3B cells, there was no significant change induced in the expression of SNRPFP1 transcript. And following the knock-out of miR-126-5p in HCC cells, the ability of cell proliferation was recovered through CCK8 assay and the Flow cytometry detection for the cell cycle (Fig. 5C,D). Simultaneously, the apoptosis rates went down too (Fig. 5E). Upon this, we suggested that the SNRPFP1 transcript played an efficient role in promoting HCC cell growth via the molecular sponge way.

## Discussion

HCC composes over 90% of the liver malignancies and leads to tough lethal outcomes, and is characterized by high tumor heterogeneity with robust invasion and migration ability^20,21^. Innovation strategies for targeting particular genes are one of the critical approaches to breaking through the bottleneck of the rapid progress, high recurrence, and dismal overall survival of HCC. However, the intensive intracellular mechanisms promoting HCC leave us largely unknown.

It has been long since the pseudogenes were regarded as ‘relics of evolution’ with no definite function^22^. Whilst, the accumulating evidence in current years verified the existence of pseudogene-derived transcripts and the related proteins, accompanied by the important roles of pseudogenes in transcriptional and post-transcriptional processes concerning human malignancies were gradually discovered^23^.

The ceRNA effect is an important function of lncRNAs and was prompted on basis of the hypothesis that the RNA products can cross-talk with lncRNA or circRNA by competitively binding to shared miRNAs and sequentially impact physiological and pathological processes, including tumorigenesis and progress^24,25^. As the pseudogene-derived RNAs are kinds of the majority components of lncRNAs, it is similar to those common lncRNAs, which can function as critical modulators in and development of human cancer by ceRNA mechanism via sponging miRNAs, which is supported by increasing findings^26^.

According to the online database LCLE we constructed in our recent study, SNRPFP1 is screened out as a pseudogene barely transcripted in normal liver tissues, but extremely activated in transcription in HCC tumors. And the corresponding parental gene, SNRPF was explored at the same time. SNRPF is a protein-coding gene, playing a key role in the alternative splicing process by building up the core component of the spliceosomal small nuclear ribonucleoproteins (snRNPs)^27,28^. In other words, dysregulation of SNRPF may potentially influence the development and progress of cancer by generating different isoforms from the pre-matured mRNAs in an alternative splicing way^29,30^. According to the literature, the independent report on SNRPF in cancers, including HCC, is limited. And much less information on its pseudogene has been reported. On this point, firstly by exploring the TCGA database and the real patients’ specimens in our study, we found that SNRPF is significantly elevated in HCC, and SNRPF1 presents a co-expression profile with the parental gene in both HCC tissues and cell lines, which prompted some significance of this gene. We wonder if the pseudogene-derived SNRPFP1 transcript exerts a definite function in HCC.

As one of the quite unknown gene products, SNRPFP1 is barely expressed in normal liver tissues and cells. However, most of the specimens of the 87 HCC cases showed a highly expressed SNRPFP1 transcript, and the rest presented relatively lower but detectable expression. Simultaneously, highly expressed SNRPFP1 was significantly correlated with the clinicopathological features of the patients, such as larger tumor size, higher serum AFP quantity, more frequency of later stages of the tumor, and the severe tumor invasiveness either intrahepatic or vascular. Moreover, the K-M plot also suggested an inclination of poor overall survival with the anomalous activation of pseudogene SNRPFP1 in HCC.

Whether the SNRPFP1 transcript is functional as a definite effector or is merely activated transcriptionally in HCC has not been reported. In this study, we depleted the highly expressed SNRPFP1 transcript, but not the parental SNRPFP1, in two HCC cell lines. Interestingly, the cell proliferation of the HCC cells was significantly impaired, which indicated the arrest of the cell cycle at the G0/S stages and the delay of tumor cell growth. Since no significant change was induced on SNRPF, we believe that SNRPFP1 could independently influence HCC cell functions. Similarly, the ability of apoptosis resistance in HCC cells was also suppressed, and abrogation of SNRPFP1 significantly elevated the number of apoptosis cells in HCC, which further supported the transcript of SNRPFP1 as an effector on HCC tumor growth. Moreover, the significant repression of cell mobility in both migration and invasion assay proposed SNRPFP1 transcript as an enhancer of tumor progress with a high risk of metastasis and local invasion.

Wondering the exact mechanism of SNRPFP1 transcript in promoting HCC progress, we investigated the possibility of the ceRNA effect implemented by this pseudogene-derived transcript. MiR-126-5p is a classic miRNA, which had been reported mainly plays the tumor-suppressive role in multiple malignancies. For instance, in breast cancer, miR-126-5p targets the PIK3R2 mRNA and sequentially suppressed the resistance to trastuzumab in tumor cells via degenerating PIK3R2 expression^31^. In gastric, miR-126 has been validated to extremely decreased and suppress tumor mobility through targeting multiple targets, like CRKL and SLC7A5^32,33^. For liver cancer, the function of miR-126-5p seems controversial but lacks adequate evidence and literature reports. In our study, we observed a significant decrease of miR-126-5p in both HCC tumor tissues and cell lines, which is supposed a trend of abrogation in HCC. Following, the significant ascent of miR-126-5p in HCC cells with the treatment of SNRPFP1 depletion strongly suggested a regulating mechanism between them. On basis of this, the seed sequence of miR-126-5p was detected, and we predicted a potential binding site on the sequence of SNRPFP1 transcript specifically interacting with miR-126-5p. The dual-luciferase reporter assay then validated the direct interaction between these two ncRNAs. And interestingly, by knock-down miR-126-5p in the HCC cells depleted SNRPFP1, the suppressed cell proliferation, and apoptosis resistance were remarkably recovered. And on this point, we tend to define miR-126-5p as a tumor-suppressive miRNA, similar to its description for the other tumors. However, the exact mechanism suppressing HCC growth by miR-126-5p needs intensive exploration.

In summary, our study innovatively discovered the activation of pseudogene SNRPFP1 transcription in HCC, and validated the adverse correlation between SNRPFP1 transcript and outcomes in HCC patients. SNRPFP1 transcript potently facilitates HCC cell growth and motility in vitro, and the ceRNA effect of it on miR-126-5p seems to be the critical mechanism. We suppose that SNRPFP1/miR-126-5p axis might be the potential and hopeful target for HCC prevention and therapeutic strategy, even though there is still much detail for investigation.

## Fundings

This study was kindly supported by grants from the following: National Natural Science Foundation of China (No. 82172900; No. 81602544); Shanghai Pujiang Talent Project (No. 18PJD029); Research physician project from Shanghai Jiao Tong University School of Medicine (No. 20191901).

## Supplementary Information

Supplementary Information 1.

Supplementary Information 2.

Supplementary Information 3.

## Data Availability

Data from this study is available on request from the authors by contacting Junqing Wang (E-mail: wangjunqingmd@hotmail.com).

## References

[1] Llovet JM (2021). Hepatocellular carcinoma. Nat. Rev. Dis. Primers.

[2] Pinero F, Dirchwolf M, Pessoa MG (2020). Biomarkers in hepatocellular carcinoma: Diagnosis prognosis and treatment response assessment. Cells.

[3] Siegel RL, Miller KD, Jemal A (2019). Cancer statistics, 2019. CA.

[4] Chan JJ, Tay Y (2018). Noncoding RNA: RNA regulatory networks in cancer. Int. J. Mol. Sci..

[5] Peng WX, Koirala P, Mo YY (2017). LncRNA-mediated regulation of cell signaling in cancer. Oncogene.

[6] Statello L, Guo CJ, Chen LL, Huarte M (2021). Gene regulation by long non-coding RNAs and its biological functions. Nat. Rev. Mol. Cell Biol..

[7] Uszczynska-Ratajczak B, Lagarde J, Frankish A, Guigo R, Johnson R (2018). Towards a complete map of the human long non-coding RNA transcriptome. Nat. Rev. Genet..

[8] Kim MS (2014). A draft map of the human proteome. Nature.

[9] Lister NC, Johnsson P, Waters PD, Morris KV (2021). Pseudogenes: A novel source of trans-acting antisense RNAs. Methods Mol. Biol..

[10] Sasidharan R, Gerstein M (2008). Genomics: Protein fossils live on as RNA. Nature.

[11] Thomson DW, Dinger ME (2016). Endogenous microRNA sponges: Evidence and controversy. Nat. Rev. Genet..

[12] Wang K (2019). Androgen receptor regulates ASS1P3/miR-34a-5p/ASS1 signaling to promote renal cell carcinoma cell growth. Cell Death Dis..

[13] Xie C (2019). A hMTR4-PDIA3P1-miR-125/124-TRAF6 regulatory axis and its function in NF kappa B signaling and chemoresistance. Hepatology.

[14] Poliseno L (2010). A coding-independent function of gene and pseudogene mRNAs regulates tumour biology. Nature.

[15] Zhang R (2017). Long non-coding RNA PTENP1 functions as a ceRNA to modulate PTEN level by decoying miR-106b and miR-93 in gastric cancer. Oncotarget.

[16] Karreth FA (2015). The BRAF pseudogene functions as a competitive endogenous RNA and induces lymphoma in vivo. Cell.

[17] Hao F (2020). Pseudogene AKR1B10P1 enhances tumorigenicity and regulates epithelial-mesenchymal transition in hepatocellular carcinoma via stabilizing SOX4. J. Cell. Mol. Med..

[18] Wang J (2020). Comprehensive network analysis reveals alternative splicing-related lncRNAs in hepatocellular carcinoma. Front. Genet..

[19] Wang N (2020). Positive feedback loop of AKR1B10P1/miR-138/SOX4 promotes cell growth in hepatocellular carcinoma cells. Am. J. Transl. Res..

[20] Llovet JM (2016). Hepatocellular carcinoma. Nat. Rev. Dis. Primers.

[21] Wong MC (2017). International incidence and mortality trends of liver cancer: A global profile. Sci. Rep..

[22] Tay Y, Rinn J, Pandolfi PP (2014). The multilayered complexity of ceRNA crosstalk and competition. Nature.

[23] Zhu Y (2018). Discovery of coding regions in the human genome by integrated proteogenomics analysis workflow. Nat. Commun..

[24] Dvinge H, Guenthoer J, Porter PL, Bradley RK (2019). RNA components of the spliceosome regulate tissue- and cancer-specific alternative splicing. Genome Res..

[25] Xiao-Jie L, Ai-Mei G, Li-Juan J, Jiang X (2015). Pseudogene in cancer: Real functions and promising signature. J. Med. Genet..

[26] An Y, Furber KL, Ji S (2017). Pseudogenes regulate parental gene expression via ceRNA network. J. Cell. Mol. Med..

[27] Bertram K (2017). Cryo-EM structure of a pre-catalytic human spliceosome primed for activation. Cell.

[28] Bertram K (2017). Cryo-EM structure of a human spliceosome activated for step 2 of splicing. Nature.

[29] Wu H (2020). Long noncoding RNA ZFAS1 promoting small nucleolar RNA-mediated 2’-O-methylation via NOP58 recruitment in colorectal cancer. Mol. Cancer.

[30] Oh JM (2020). U1 snRNP regulates cancer cell migration and invasion in vitro. Nat. Commun..

[31] Fu R, Tong JS (2020). miR-126 reduces trastuzumab resistance by targeting PIK3R2 and regulating AKT/mTOR pathway in breast cancer cells. J. Cell. Mol. Med..

[32] Feng R (2018). Down-regulated serum miR-126 is associated with aggressive progression and poor prognosis of gastric cancer. Cancer Biomark..

[33] Wang J (2016). SLC7A5 functions as a downstream target modulated by CRKL in metastasis process of gastric cancer SGC-7901 cells. PLoS ONE.

