# Supplementary material for: Pseudogene SNRPFP1 derived long non-coding RNA facilitates hepatocellular carcinoma progress in vitro by sponging tumor-suppressive miR-126-5p
Source: Sci Rep. 2022 Dec 19;12:21867. doi: 10.1038/s41598-022-24597-5 (PMC9763376; doi:10.1038/s41598-022-24597-5)
Supplement: Supplementary file 1 — Supplementary Information 1. [file 41598_2022_24597_MOESM1_ESM.docx]

**Suppl. Table. 1**

**The primers for RT-qPCR assay**

| **Genes** | **Forward** | **Reverse** |
| --- | --- | --- |
| **SNRPF** | **5’-AACTAAAAGCCGCTCCGCTC-3’** | **5’-TCTGGTGGCCAGAAAACAGAAG-3’** |
| **SNRPFP1** | **5’-TCAGTGGAGTAACAGGAAAGCC-3** | **5’-TCCTCTTCTTCCACACCTCTGA-3** |
| **miR-126-5p** | **5’-CTCAACTGGTGTCGTGGACGCGTACC-3** | **5’-ACACTCCAGCTGGGCATTATTACTTTTGG-3** |
|  |  |  |

**Suppl. Table. 2**

**The selected sequences of the predicted miR-126-5p binding site of SNRPFP1 transcript, along with the relative mutated sequences**

| **Genes** | **Sequence including the binding site**  **(202 bp)** | **Relative mutated sequence** |
| --- | --- | --- |
| **SNRPFP1** | 5’-gccaaggatggtgaaacttaagcggggaatggagtactagggctacctggtatctgtagatggctacatgaacatgcagcttgcaaatacagaagaatacatagatggagcattgtctggacatctgggtgaagttttaataaggtgtaataatgtcctttatatcagaggtgtggaagaagaggaagaagatggagaaatg-3’ | 5’-ggctacgttcgagtatcattacccgcgtaagcacttcaacgccaagcagctttgtcttgttcggttcttcatcttcctggtaggataaagacatgtaaagaaacaagcaccttagacagcagaacagcgagtactataatttacgagaatttaagacgtatttttgacacgagagcatgtacacgtacatgttcgtgtattc-3’ |
|  |  |  |

**Suppl. Table. 3: Correlation between SNRPF expression and clinicopathological features in 87 HCC specimens.**

SNRPF expression associated with clinicopathologic features in 87 HCC patients, including age, gender, tumor size, tumor stage (AJCC), tumor encapsulation, tumor microsatellite formation, vein invasion, HBsAg status, AFP level, and liver cirrhosis. Statistically, significance was assessed by Fisher’s exact test.

| **Clinicopathological parameters** | **SNRPFP1 transcript level** | | ***P**** |
| --- | --- | --- | --- |
|  | Low (n=12) | High(n=75) |  |
| **Age (years)**  **≤50**  **＞50** |  |  |  |
|  | **8** | **38** | **0.363** |
|  | **4** | **37** |  |
| **Gender**  **Male**  **Female** |  |  |  |
|  | **8** | **38** | **0.362** |
|  | **4** | **37** |  |
| **Diameter (cm)**  **≤5**  **＞5** |  |  |  |
|  | **10** | **33** | **0.014** |
|  | **2** | **42** |  |
| **TNM stage**  **I～II**  **III～IV** |  |  |  |
|  | **8** | **24** | **0.027** |
|  | **4** | **51** |  |
| **Tumor encapsulation**  **Absent**  **Present** |  |  |  |
|  | **7** | **29** | **0.222** |
|  | **5** | **46** |  |
| **Tumor microsatellite formation**  **Absent**  **Present** |  |  |  |
|  | **9** | **21** | **0.009** |
|  | **3** | **44** |  |
| **Venous invasion**  **No**  **Yes** |  |  |  |
|  | **6** | **26** | **0.345** |
|  | **6** | **49** |  |
| **HBsAg**  **Negative**  **Positive** |  |  |  |
|  | **3** | **9** | **0.362** |
|  | **9** | **65** |  |
| **AFP(ng/ml)**  **≤400**  **＞400** |  |  |  |
|  | **7** | **13** | **0.005** |
|  | **5** | **62** |  |
| **Cirrhosis**  **Absent**  **Present** |  |  |  |
|  | **5** | **5** | **0.004** |
|  | **7** | **70** |  |

**Supplementary Figure Legends：**

**Suppl. Fig. 1.**

**Suppl. Fig. 1. Expression profile of the parental gene SNRPF, and the overall survival correlated with pseudogene**

**
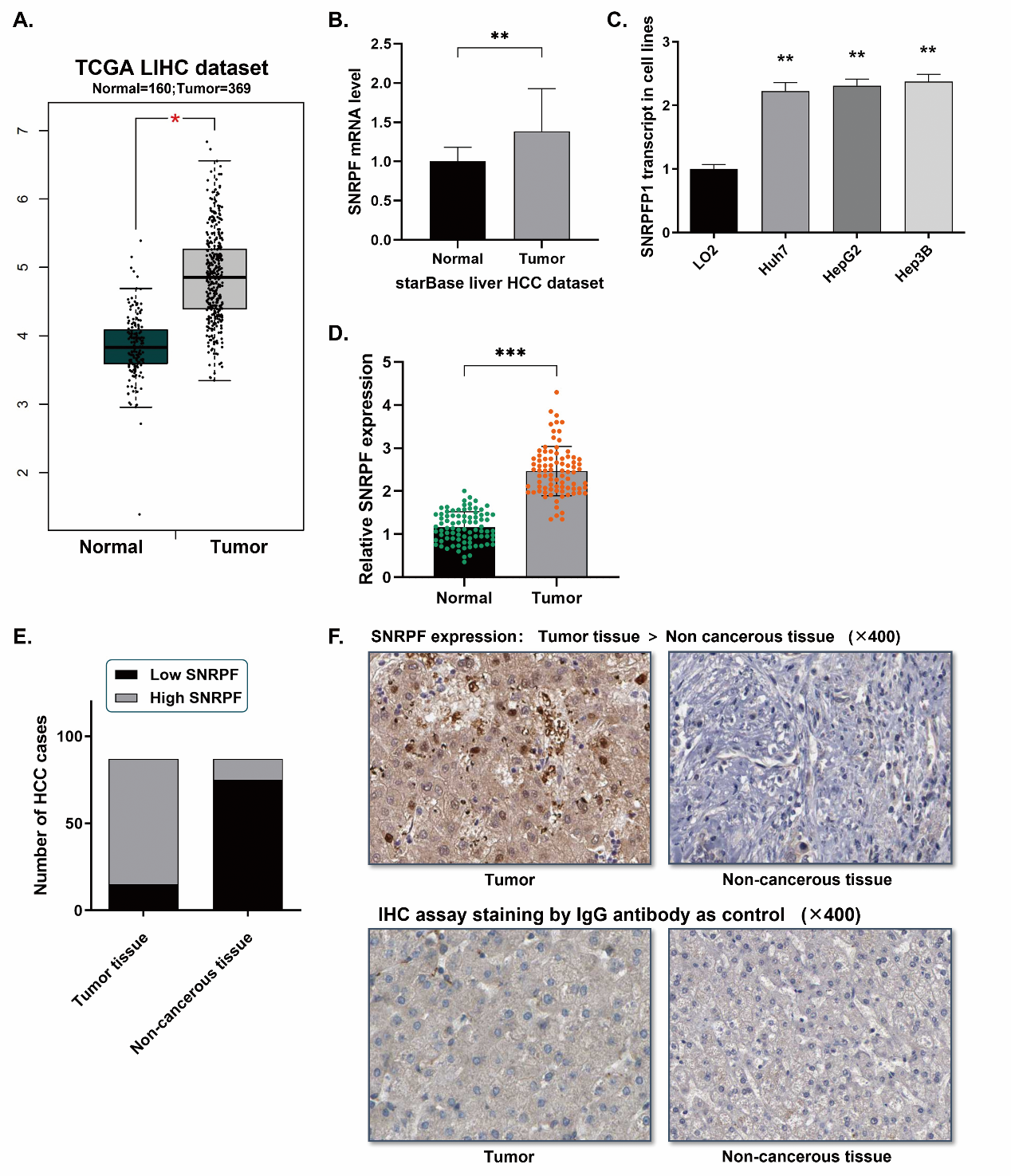
**

**A.** The expression profile of SNRPF in HCC tumor tissues, compared with the normal liver tissues, according to the analysis of liver hepatocellular carcinoma dataset from the TCGA database (**P*＜0.05). **B.** The expression profile of SNRPF in HCC tumor tissues, compared with the normal liver tissues, according to the analysis of liver hepatocellular carcinoma dataset from the starBase database (***P*＜0.01). **C.** The RT-qPCR assay indicated that SNRPF is highly expressed in the three HCC cell lines, in comparison with the control LO2 cells (***P*＜0.01). **D.** The expression level of SNRPF mRNA was detected by the RT-qPCR assay in the 87 real patients specimens. SNRPF was significantly higher in tumor tissues than in the non-cancerous liver tissues (****P*＜0.001). **E.** Statistic of the number of cases concerning the expression of SNRPF in HCC specimens. SNRPF is highly expressed in most of the tumor tissues (72/87), and only a small portion of the adjacent non-cancerous tissues presented relatively higher SNRPF expression (12/87) (***P*＜0.01). **F.** Representative graph of immunohistochemistry analysis (400🞩) of the HCC cases. The IgG antibody was used for staining the specimens as a control. SNRPF expression in tumor specimens was significantly higher than in adjacent non-cancerous tissues. **G.** Moreover, the 2-year overall survival of the 87 real HCC patients collected from our medical center demonstrates a poor outcome of the overall survival in the patients with a highly expressed transcript of SNRPF1, which is the pseudogene of SNRPF (***P*＜0.01).
