# Supplementary material for: Pseudogene SNRPFP1 derived long non-coding RNA facilitates hepatocellular carcinoma progress in vitro by sponging tumor-suppressive miR-126-5p
Source: Sci Rep. 2022 Dec 19;12:21867. doi: 10.1038/s41598-022-24597-5 (PMC9763376; doi:10.1038/s41598-022-24597-5)
Supplement: Supplementary file 2 — Supplementary Information 2. [file 41598_2022_24597_MOESM2_ESM.doc]

**STR Genotype Test Report**

**Sample name: HEP 3B**

**Method:** DNA was extracted with the genome extraction kit of Axygen, amplified with the 20 STR amplification scheme, and the STR site and sex gene Amelogenin were detected on ABI 3730XL genetic analyzer.

**Results:** The DNA typing of this cell line found an exact matching cell line in the cell line search. The DSMZ database showed that the cell name was HEP-3B, and the cell number corresponded to 93. No multiple alleles were found in this cell line. Cell matching value: 1.0

**Comparison and analysis of STR database:** The genotyping results of STR sites and Amelogenin sites of the cells to be tested were compared with the STR data of 2455 cell lines included in ExPASY, ATCC, DSMZ, JCRB and RIKEN databases. If the cells to be tested were not included in the above cell banks or this is a new cell line established by ourselves, it would be impossible to compare. Users need to compare with other databases according to the cell typing results.

**Typing results:**

| Loci | STR information of submitted cells | | | Cell bank cell STR information | | |
| --- | --- | --- | --- | --- | --- | --- |
|  | Cell：HEP-3B | | | Cell bank：HEP-3B | | |
|  | Allele1 | Allele2 | Allele3 | Allele1 | Allele2 | Allele3 |
| D5S818 | 13 | 13 |  | 13 | 13 |  |
| D13S317 | 12 | 14 |  | 12 | 14 |  |
| D7S820 | 8 | 10 |  | 8 | 10 |  |
| D16S539 | 10 | 10 |  | 10 | 10 |  |
| VWA | 17 | 17 |  | 17 | 17 |  |
| TH01 | 6 | 7 |  | 6 | 7 |  |
| AMEL | X | X |  | X | X |  |
| TPOX | 9 | 9 |  | 9 | 9 |  |
| CSF1PO | 8 | 8 |  | 8 | 8 |  |
| D12S391 | 17 | 17 |  |  |  |  |
| FGA | 18 | 18 |  |  |  |  |
| D2S1338 | 21 | 25 |  |  |  |  |
| D21S11 | 30 | 31 |  |  |  |  |
| D18S51 | 20 | 20 |  |  |  |  |
| D8S1179 | 12 | 12 |  |  |  |  |
| D3S1358 | 15 | 15 |  |  |  |  |
| D6S1043 | 12 | 17 |  |  |  |  |
| PENTAE | 5 | 16 |  |  |  |  |
| D19S433 | 12.2 | 14 |  |  |  |  |
| PENTAD | 12 | 14 |  |  |  |  |

**Typing map:**


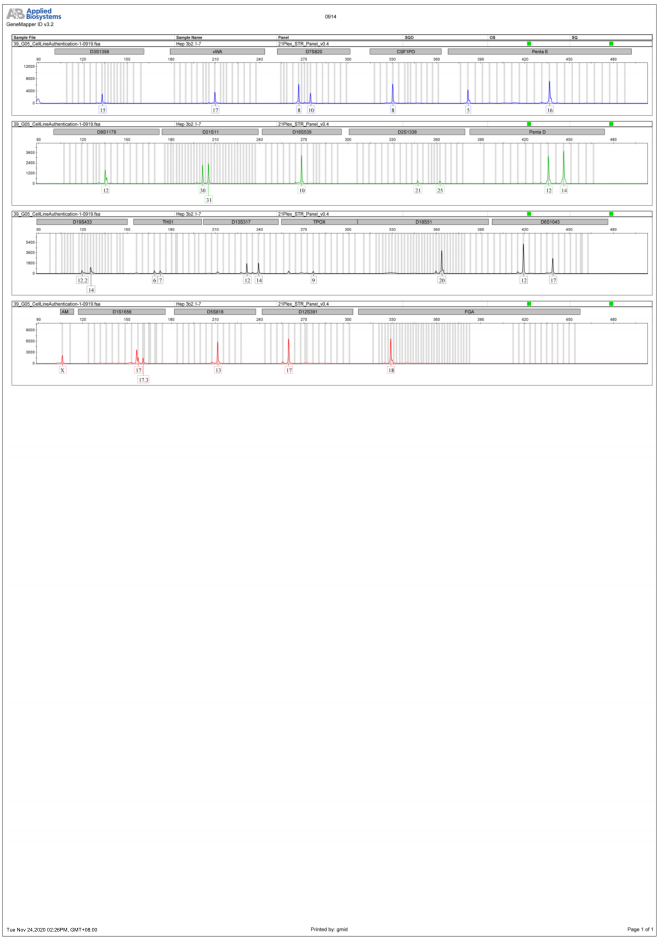


**Remarks:**

1. According to the cell STR identification standard formulated by the International Commission for Cell Identification (ICLAC), if the matching degree is ≥ 80%, the cell line can be considered correct; if the matching degree is ＜ 80%, the source of the cell line needs to be suspected.
2. Real PCR bands with effective peak positions of the map, small peaks and non-specific bands are ignored in the calculation.
3. National experimental cell resource sharing platform, database entry http://www.cellresource.cn /。

**STR Genotype Test Report**

**Sample name: HUH-7**

**Method:** DNA was extracted with the genome extraction kit of Axygen, amplified with the 20 STR amplification scheme, and the STR site and sex gene Amelogenin were detected on ABI 3730XL genetic analyzer.

**Results:** The DNA typing of this cell line found an exact matching cell line in the cell line search. The DSMZ database shows that the cell name is HuH-7, and the cell number corresponds to JCRB0403. No multiple alleles were found in this cell line. Cell matching value: 1.0

**Comparison and analysis of STR database:** The genotyping results of STR sites and Amelogenin sites of the cells to be tested were compared with the STR data of 2455 cell lines included in ExPASY, ATCC, DSMZ, JCRB and RIKEN databases. If the cells to be tested were not included in the above cell banks or this is a new cell line established by ourselves, it would be impossible to compare. Users need to compare with other databases according to the cell typing results.

**Typing results:**

| Loci | STR information of submitted cells | | | Cell bank cell STR information | | |
| --- | --- | --- | --- | --- | --- | --- |
|  | Cell：HuH-7 | | | Cell bank：HuH-7 | | |
|  | Allele1 | Allele2 | Allele3 | Allele1 | Allele2 | Allele3 |
| D5S818 | 12 | 12 |  | 12 | 12 |  |
| D13S317 | 10 | 11 |  | 10 | 11 |  |
| D7S820 | 11 | 11 |  | 11 | 11 |  |
| D16S539 | 10 | 10 |  | 10 | 10 |  |
| VWA | 16 | 18 |  | 16 | 18 |  |
| TH01 | 7 | 7 |  | 7 | 7 |  |
| AMEL | X | X |  | X | X |  |
| TPOX | 8 | 11 |  | 8 | 11 |  |
| CSF1PO | 11 | 11 |  | 11 | 11 |  |
| D12S391 | 20 | 21 |  |  |  |  |
| FGA | 22 | 23 |  |  |  |  |
| D2S1338 | 19 | 19 |  |  |  |  |
| D21S11 | 30 | 30 |  |  |  |  |
| D18S51 | 15 | 15 |  |  |  |  |
| D8S1179 | 14 | 14 |  |  |  |  |
| D3S1358 | 15 | 15 |  |  |  |  |
| D6S1043 | 13 | 15 |  |  |  |  |
| PENTAE | 11 | 11 |  |  |  |  |
| D19S433 | 13 | 14 |  |  |  |  |
| PENTAD | 12 | 12 |  |  |  |  |
| D1S1656 | 16 | 16 |  |  |  |  |

**Typing map:**


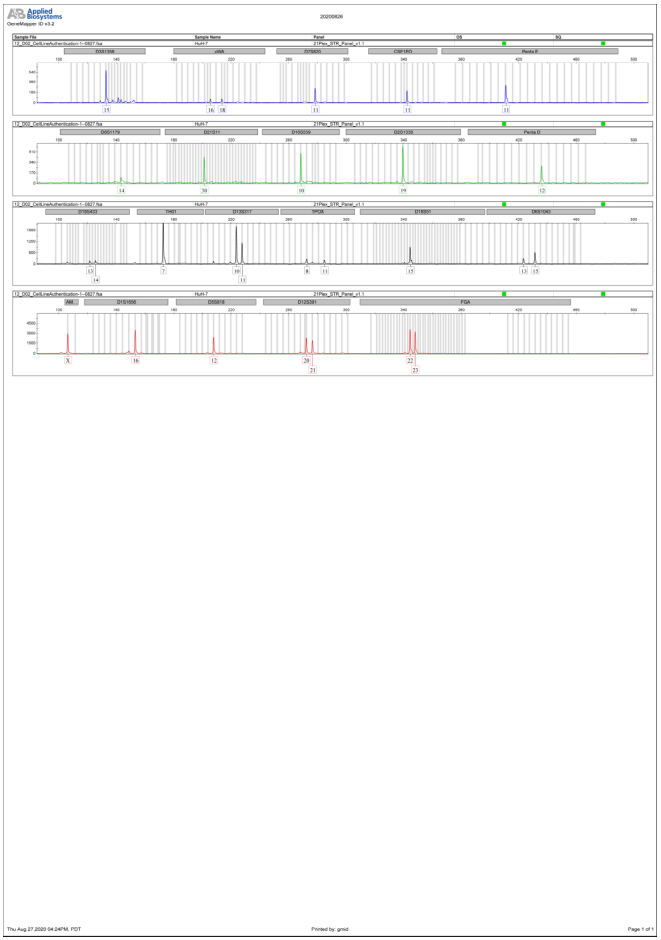


**Remarks:**

1. According to the cell STR identification standard formulated by the International Commission for Cell Identification (ICLAC), if the matching degree is ≥ 80%, the cell line can be considered correct; if the matching degree is ＜ 80%, the source of the cell line needs to be suspected.
2. Real PCR bands with effective peak positions of the map, small peaks and non-specific bands are ignored in the calculation.
3. National experimental cell resource sharing platform, database entry http://www.cellresource.cn /。

**STR Genotype Test Report**

**Sample name: HEPG2**

**Method:** DNA was extracted with the genome extraction kit of Axygen, amplified with the 20 STR amplification scheme, and the STR site and sex gene Amelogenin were detected on ABI 3730XL genetic analyzer.

**Results:** The DNA typing of this cell line found an exact matching cell line in the cell line search. The DSMZ database shows that the cell name is HEP-G2, and the cell number corresponds to 180. No multiple alleles were found in this cell line. Cell matching value: 1.0

**Comparison and analysis of STR database:** The genotyping results of STR sites and Amelogenin sites of the cells to be tested were compared with the STR data of 2455 cell lines included in ExPASY, ATCC, DSMZ, JCRB and RIKEN databases. If the cells to be tested were not included in the above cell banks or this is a new cell line established by ourselves, it would be impossible to compare. Users need to compare with other databases according to the cell typing results.

**Typing results:**

| Loci | STR information of submitted cells | | | Cell bank cell STR information | | |
| --- | --- | --- | --- | --- | --- | --- |
|  | Cell：HEPG2 | | | Cell bank：HEP-G2 | | |
|  | Allele1 | Allele2 | Allele3 | Allele1 | Allele2 | Allele3 |
| D5S818 | 11 | 12 |  | 11 | 12 |  |
| D13S317 | 9 | 13 |  | 9 | 13 |  |
| D7S820 | 10 | 10 |  | 10 | 10 |  |
| D16S539 | 12 | 13 |  | 12 | 13 |  |
| VWA | 17 | 17 |  | 17 | 17 |  |
| TH01 | 9 | 9 |  | 9 | 9 |  |
| AMEL | X | Y |  | X | Y |  |
| TPOX | 8 | 9 |  | 8 | 9 |  |
| CSF1PO | 10 | 11 |  | 10 | 11 |  |
| D12S391 | 21 | 25 |  |  |  |  |
| FGA | 22 | 25 |  |  |  |  |
| D2S1338 | 19 | 20 |  |  |  |  |
| D21S11 | 29 | 31 |  |  |  |  |
| D18S51 | 13 | 14 |  |  |  |  |
| D8S1179 | 15 | 16 |  |  |  |  |
| D3S1358 | 15 | 16 |  |  |  |  |
| D6S1043 | 13 | 13 |  |  |  |  |
| PENTAE | 15 | 20 |  |  |  |  |
| D19S433 | 15.2 | 15.2 |  |  |  |  |
| PENTAD | 9 | 13 |  |  |  |  |
| D1S1656 | 11 | 12 |  |  |  |  |

**Typing map:**


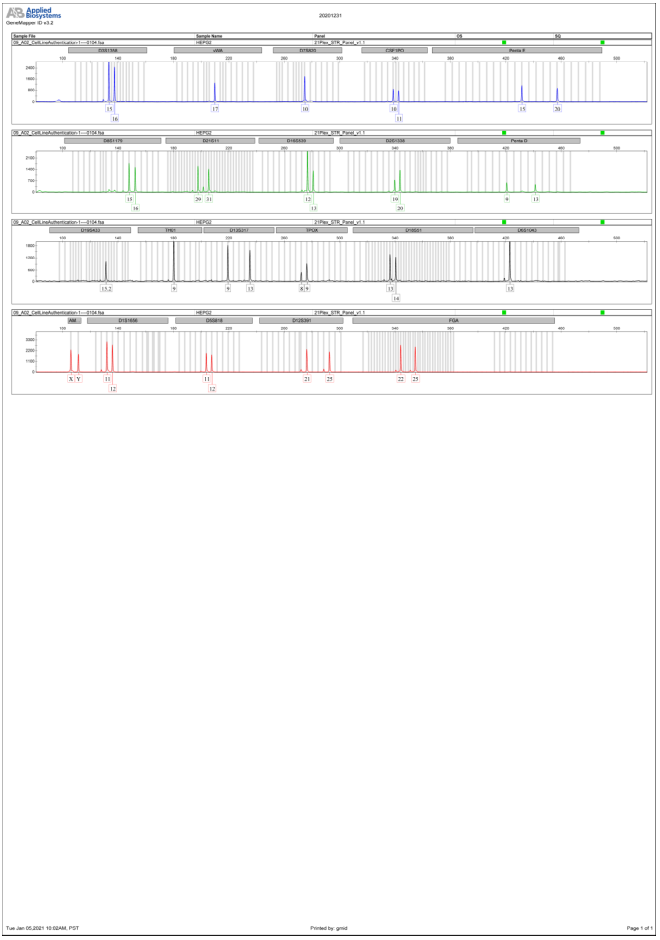


**Remarks:**

1. According to the cell STR identification standard formulated by the International Commission for Cell Identification (ICLAC), if the matching degree is ≥ 80%, the cell line can be considered correct; if the matching degree is ＜ 80%, the source of the cell line needs to be suspected.
2. Real PCR bands with effective peak positions of the map, small peaks and non-specific bands are ignored in the calculation.
3. National experimental cell resource sharing platform, database entry http://www.cellresource.cn /。

**STR Genotype Test Report**

**Sample name: HL-7702 (L-02)**

**Method:** DNA was extracted with the genome extraction kit of Axygen, amplified with the 20 STR amplification scheme, and the STR site and sex gene Amelogenin were detected on ABI 3730XL genetic analyzer.

**Results:** The DNA typing of this cell line found a basically matched cell line in the cell line search. The DSMZ database shows that the cell name is L-02, and the cell number corresponds to CVCL_ 6926。 No multiple alleles were found in this cell line. Cell matching value: 0.96.

Problematic cell line: Contaminated. Shown to be a HeLa derivative (PubMed=26116706). Originally thought to originate from a normal fetal liver.


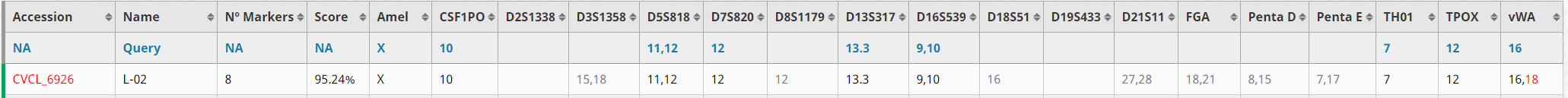


**Comparison and analysis of STR database:** The genotyping results of STR sites and Amelogenin sites of the cells to be tested were compared with the STR data of 2455 cell lines included in ExPASY, ATCC, DSMZ, JCRB and RIKEN databases. If the cells to be tested were not included in the above cell banks or this is a new cell line established by ourselves, it would be impossible to compare. Users need to compare with other databases according to the cell typing results.

**Typing results:**

| Loci | STR information of submitted cells | | | Cell bank cell STR information | | |
| --- | --- | --- | --- | --- | --- | --- |
|  | Cell：HL-7702 | | | Cell bank：L-02 | | |
|  | Allele1 | Allele2 | Allele3 | Allele1 | Allele2 | Allele3 |
| D5S818 | 11 | 12 |  | 11 | 12 |  |
| D13S317 | 13.3 | 13.3 |  | 13.3 | 13.3 |  |
| D7S820 | 12 | 12 |  | 12 | 12 |  |
| D16S539 | 9 | 10 |  | 9 | 10 |  |
| VWA | 16 | 16 |  | 16 | 18 |  |
| TH01 | 7 | 7 |  | 7 | 7 |  |
| AMEL | X | X |  | X | X |  |
| TPOX | 12 | 12 |  | 12 | 12 |  |
| CSF1PO | 10 | 10 |  | 10 | 10 |  |
| D12S391 | 20 | 20 |  |  |  |  |
| FGA | 18 | 21 |  |  |  |  |
| D2S1338 | 17 | 17 |  |  |  |  |
| D21S11 | 27 | 28 |  |  |  |  |
| D18S51 | 16 | 16 |  |  |  |  |
| D8S1179 | 12 | 12 |  |  |  |  |
| D3S1358 | 15 | 18 |  |  |  |  |
| D6S1043 | 18 | 18 |  |  |  |  |
| PENTAE | 7 | 17 |  |  |  |  |
| D19S433 | 13 | 13 |  |  |  |  |
| PENTAD | 8 | 15 |  |  |  |  |
| D1S1656 | 12 | 15 |  |  |  |  |

**Typing map:**


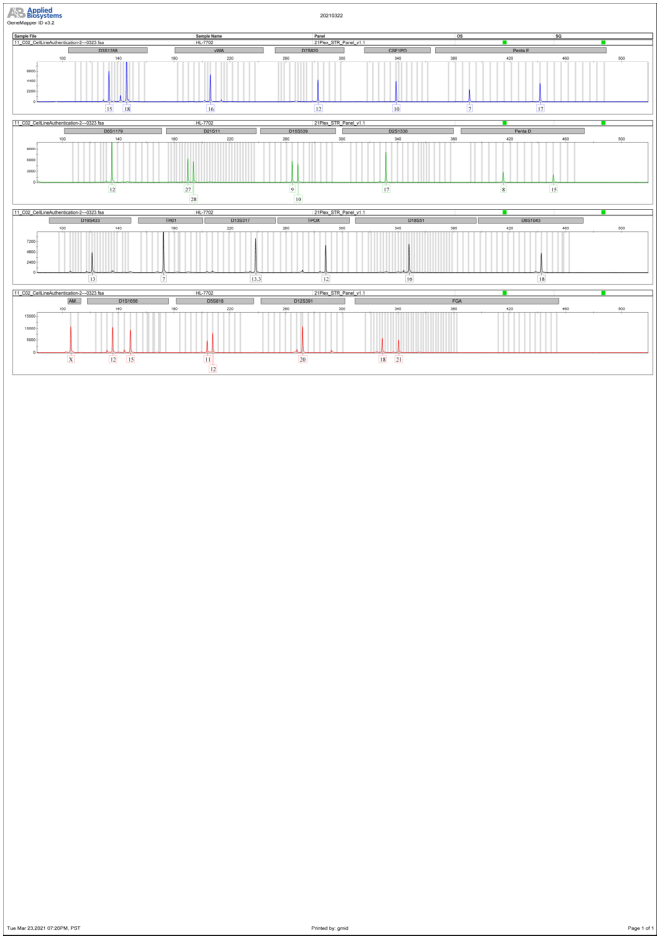


**Remarks:**

1. According to the cell STR identification standard formulated by the International Commission for Cell Identification (ICLAC), if the matching degree is ≥ 80%, the cell line can be considered correct; if the matching degree is ＜ 80%, the source of the cell line needs to be suspected.
2. Real PCR bands with effective peak positions of the map, small peaks and non-specific bands are ignored in the calculation.
3. National experimental cell resource sharing platform, database entry http://www.cellresource.cn /。
